# Preparation and Application of Polymer Nanocomposites

**DOI:** 10.3390/nano13040657

**Published:** 2023-02-08

**Authors:** Teresa Cuberes

**Affiliations:** Department of Applied Mechanics and Project Engineering, Mining and Industrial Engineering School of Almaden, University of Castilla-La Mancha, Plaza Manuel Meca 1, 13400 Almadén, Spain; teresa.cuberes@uclm.es

The incorporation of nanomaterials into polymer matrices opens new avenues for the development of advanced materials with unique novel properties and impact in many different fields. A thorough understanding of how nanofillers affect the structural conformation of the polymer matrix at different hierarchical levels; how to control the dispersion, aggregation, and organization of nanofillers within the matrix; and how to tune and monitor the interfacial properties still remains a challenge. The current issue includes interesting examples of the preparation of polymer nanocomposites for different applications:

Structural nanocomposite materials and their mechanical properties are considered in [1,2]. In [1], the benefits of the incorporation of nanoclay into polymer-modified asphalt are investigated; intercalated or exfoliated nanoclay structures hinder the migration of insoluble additive of polymers, hindering phase separation, and improving its storage stability, which is a major concern in asphalt modification technologies. [2] studies the effect of adding borpolymer to ultra-high-molecular-weight polyethylene, which does not chemically interact with the matrix but acts as a reinforcing filler, modifying the supramolecular structure of the matrix, and hence its deformation and strength responses.

Nanocomposites for electronics and nanofabrication are considered in [3,4]. In [3], an innovative resist based on CuO/polymethyl methacrylate (PMMA) is developed; the CuO nanostructures with typical sizes of 10–30 nm provide functionality to the resist, which can be patterned by electron beam lithography. [4] reviews the dielectric-thickness dependence of the electric breakdown strength, which is a critical parameter in the design of solid insulation structures, discussing the responsible mechanisms for the thickness effect.

Nanocomposite-based nanosensors and nanogenerators are considered in [5,6]. In [5], a flexible capacitive pressure sensor is proposed, formed by a nanocomposite dielectric layer of porous polydimethyl siloxane elastomer and ZnO nanowire, which maintains its response over a wide dynamic range from 1 Pa to 50 KPa, almost covering the entire tactile-pressure range. Ref. [6] develops triboelectric energy harvesters based on polyvinylidene fluoride (PVDF) and polyvinyl amide (PA) fibrous composites, evaluating their dielectric and triboelectric responses.

Eventually, nanocomposites for biomedical applications are considered in [7,8]. In [7], poly(ε-caprolactone) nanocomposites with multiwall nanotubes and electrospan carbon fibers are investigated to improve cell adhesion; human osteoblast-like cells were successfully developed on the nanocomposites, with their interactions being conveniently monitored from the early stages through Raman microspectroscopy. Ref. [8] studies organic–inorganic ureasil–polyether hybrid matrices for drug delivery, using dexamethasone acetate as a model drug; ultrasonic force microscopy reveals a structural organization at ureasil–poly(propylene oxide)400 films in which clusters of correlated siloxane nodes form beads that align into strands, gathering into hybrid polymer ropes on the film surface, which impacts the behavior of the matrix as a drug host.
